# Comparison of the Therapeutic Effects of Rebamipide and Diquafosol on Apoptotic Damage of the Ocular Surface in Dry Eyes

**DOI:** 10.3390/antiox14070780

**Published:** 2025-06-25

**Authors:** Enying Jiang, Hui Jin, Jingting Liu, Hyun Jee Kim, Hee Su Yoon, Ji Suk Choi, Jayoung Moon, Hoon-In Choi, Hyeon-Jeong Yoon, Kyung Chul Yoon

**Affiliations:** 1 Department of Ophthalmology, Chonnam National University Medical School and Hospital, Gwangju 61469, Republic of Korea; enyeong99925@gmail.com (E.J.);; 2 Department of Biomedical Sciences and Centers for Creative Biomedical Scientists at Chonnam National University, Gwangju 61469, Republic of Korea

**Keywords:** dry eye disease, apoptosis, oxidative stress, rebamipide, diquafosol

## Abstract

Dry eye disease (DED) is characterized by tear film instability and oxidative stress-induced epithelial damage. This study was conducted to compare the therapeutic effects of 2% rebamipide (REB) and 3% diquafosol (DQS) on oxidative stress-related apoptotic damage of the ocular surface in DED. Human corneal epithelial cells (HCECs) were exposed to hyperosmotic stress in vitro and treated with REB or DQS. Cell viability and cleaved caspase-3 expression were evaluated using the MTT assay and Western blotting. DED was induced in vivo in C57BL/6 mice using subcutaneous scopolamine injection. Thereafter, the mice were assigned to normal control (NC), dry eye (DE), DQS-treated (DQS), or REB-treated (REB) groups. Clinical evaluations, including measurement of tear film break-up time, corneal smoothness, and the lipid layer, were performed on days 7 and 14. In addition, CD4^+^ IFN-γ^+^ T cells, inflammatory cytokines, reactive oxygen species (ROS), lipid peroxidation markers, and corneal apoptosis were analyzed on day 14. Glycocalyx integrity and goblet cell density were also evaluated. The results indicate that REB improved HCEC survival and suppressed cleaved caspase-3 expression more effectively than DQS (*p* < 0.05). Both treatments improved clinical outcomes in the murine dry eye model; however, REB showed superior efficacy in reducing ROS levels, lipid peroxidation, and apoptosis, and in preserving corneal glycocalyx integrity and conjunctival goblet cell density. These findings highlight the therapeutic potential and protective effects of REB against oxidative stress-related damage and apoptosis in DED.

## 1. Introduction

Dry eye disease (DED) is a multifactorial ocular surface disorder characterized by loss of tear film homeostasis and its associated ocular symptoms. Tear film instability and excessive evaporation lead to tear hyperosmolarity, triggering inflammation and cytokine release. This inflammatory response weakens the ocular surface barrier, resulting in goblet cell loss and epithelial apoptosis. These changes further destabilize the tear film, creating a vicious cycle that progressively worsens the disease [1,2,3,4].

Oxidative stress contributes significantly to ocular surface damage in DED. Oxidative stress occurs as a result of an imbalance between the generation of reactive oxygen species (ROS) and the antioxidant defense system. Accumulation of excessive ROS disrupts cellular homeostasis, triggers lipid peroxidation, and activates pro-inflammatory pathways, ultimately accelerating corneal epithelial apoptosis and worsening ocular surface damage [5,6,7].

Diquafosol (DQS), a P2Y2 receptor agonist, improves tear film stability and ocular surface wetting by promoting the secretion of aqueous fluid and mucin from conjunctival epithelial and goblet cells [8]. In addition, studies have shown that DQS increases goblet cell density and prolongs tear film break-up time (TBUT) [9]. Rebamipide (REB), a mucin secretagogue, has recently emerged as another promising treatment for DED. Beyond enhancing tear film stability, REB exhibits antioxidant properties, promotes glycocalyx mucin production, and supports epithelial integrity, thereby reinforcing the tear film. Additionally, its anti-inflammatory effects contribute to the protection of the ocular surface [10,11,12].

Previous studies have been conducted to compare the efficacy of REB and DQS in tear film stabilization and the suppression of inflammation [13,14]. However, the effects of both therapeutic agents on oxidative stress and apoptosis remain largely unexplored. Given the critical role of ROS-induced oxidative stress in corneal epithelial damage and the progression of DED, understanding how REB and DQS attenuate oxidative damage and suppress apoptosis is essential.

Thus, the aim of this study was to investigate the therapeutic potential of REB and DQS in mitigating oxidative stress and apoptosis on the ocular surface in dry eyes. Specifically, we examined the protective effects of REB and DQS against hyperosmotic stress-induced apoptosis in human corneal epithelial cells (HCECs), and assessed their therapeutic efficacy by examining inflammation, oxidative damage, epithelial apoptosis, and clinical outcomes in a murine model of DED.

## 2. Materials and Methods

### 2.1. Corneal Epithelial Cell Preparation and Treatment

Primary human corneal epithelial cells (HCECs) were obtained from donor corneas, provided by the Department of Ophthalmology, Pusan National University, Republic of Korea. The cells were cultured in a 1:1 mixture of KSF Medium supplemented with growth factors (Gibco, Waltham, MA, USA, Cat# 17005042), and DMEM/F12 (Gibco, MA, USA, Cat# 11330-032) supplemented with 10% fetal bovine serum (FBS) and 1× penicillin–streptomycin (P/S). The cultures were maintained at 37 °C in a humidified atmosphere containing 5% CO_2_. The cells were passaged at confluence, and all experiments were conducted using cells between passages 7 and 8. Hyperosmotic stress was induced by adding NaCl to the culture medium at final concentrations of 0, 50, 100, or 500 mM.

Rebamipide (REB, 2%) or diquafosol (DQS, 3%) was applied to the HCECs following the NaCl-induced hyperosmotic stress to assess their therapeutic effects. The experimental groups were categorized as follows: (1) NC group: untreated cells maintained under normal conditions; (2) NaCl group: cells exposed to 100 mM NaCl alone; (3) DQS group: cells exposed to NaCl with DQS; and (4) REB group: cells exposed to NaCl with REB treatment.

### 2.2. Analysis of Cell Viability and Morphology, and Western Blotting

HCECs were incubated with MTT (5 mg/mL) for 4 h. Formazan was solubilized in DMSO, and the absorbance at 570 nm was measured using a microplate reader (Molecular Devices, San Jose, CA, USA). The results were normalized to controls. Cell morphology was examined via phase-contrast microscopy (AXIOVERT5, Carl Zeiss, 100×, Oberkochen, Germany) [15].

Proteins were extracted using RIPA buffer (Tris-HCl, NaCl, EDTA, NP-40, SDS; pH 7.6) with protease inhibitors. Lysates were sonicated and centrifuged (15,000× *g*, 10 min, 4 °C). Band intensities were quantified by densitometry using ImageJ (Version 1.53, NIH, Bethesda, MD, USA) [16].

### 2.3. Animal Study Design

The procedures for all the animal experiments were approved by the Institutional Animal Care and Use Committee of Chonnam National University Medical School (Approval No. CNUHIACUC-24038; approved on 22 November 2024), and adhered to the ARVO Statement for the Use of Animals in Ophthalmic and Vision Research. The female C57BL/6 mice (7–8 weeks old) used in this study were maintained under standard conditions with free access to food and water.

Dry eye was induced in the mice by subcutaneous administration of scopolamine hydrobromide (0.5 mg/0.2 mL), given three times daily for 14 days, under desiccating environmental conditions (continuous airflow, 30% humidity) [17,18]. The mice were randomly assigned to four groups: (1) the normal control (NC) group: mice not subjected to desiccating stress; (2) the dry eye (DE) group: mice exposed to desiccating stress without treatment; (3) the DQS group: mice treated with 3% diquafosol (Santen Pharmaceutical Co., Ltd., Osaka, Japan); and (4) the REB group: mice treated with 2% rebamipide (Samil Pharmaceutical Co., Ltd., Seoul, Republic of Korea). Ocular instillation (2 μL/eye) was conducted four times daily at regular intervals. Clinical assessments (measurement of TBUT, corneal smoothness, and lipid layer interference) were performed on days 7 and 14. Following the clinical assessments, the mice were euthanized and their ocular tissues were collected for analysis. The analysis procedures included flow cytometry, Luminex-based cytokine assay, DCFDA assay for ROS, TUNEL staining for apoptosis, and immunofluorescence for MDA and 4-HNE. Additional evaluations involved glycocalyx staining and histological examination. The experiments were independently conducted in three replicates.

### 2.4. Clinical Evaluations

A 1 μL drop of 1% fluorescein was applied to the lower conjunctiva, and TBUT was examined under cobalt blue illumination using a slit-lamp after three natural blinks [19].

For the evaluation of corneal smoothness, a circular illumination ring from a fiberoptic source integrated into a stereoscopic microscope (SMZ1500 stereomicroscope, Nikon, Tokyo, Japan) was projected onto the corneal surface. Two masked observers scored corneal ring distortion from 0 to 5, where 0 indicated a uniform ring, 1–4 indicated increasing quadrant-wise distortion, and 5 indicated complete disruption [20].

Corneal lipid layer patterns were assessed under uniform reflected light using a stereomicroscope (SZM45TR-STL2; SOPTOP, Yongkang, China). After a spontaneous blink, a masked evaluator graded the interference images on a validated six-point scale, based on mesh density, waveform clarity, and color transition, from faint gray (grade 1) to intense blue–brown banding (grade 6) [21,22].

### 2.5. Analysis of ROS Levels and Lipid Peroxidation Markers

ROS production in ocular surface cells was quantified via CM-H2DCFDA assay (Invitrogen, Carlsbad, CA, USA) following collagenase digestion. Flow cytometric analysis (FACSCalibur, BD Biosciences, San Jose, CA, USA) was performed, and the values were normalized to control levels using CellQuest software (version 5.2.1; BD Biosciences) [23].

Imaging was carried out on a Leica DM2500 (Wetzlar, Germany) following immunostaining with antibodies against 4-HNE (5 µg/mL) and MDA (2 µg/mL). Alexa Fluor 488 (1:200) served as the secondary label, and nuclei were visualized with DAPI. The mean fluorescence intensity (MFI) was assessed for three tissue sections per sample [23].

### 2.6. Detection of Corneal Apoptosis Using the TUNEL Assay

Apoptotic cells in corneal sections were visualized using the DeadEnd™ Fluorometric kit (Promega, Madison, WI, USA) and counterstained with DAPI. Confocal images (Leica, Wetzlar, Germany) were used to quantify TUNEL-positive nuclei in the epithelium [24].

### 2.7. Measurement of T Cell Activation and Cytokine Profiles

Single-cell suspensions from corneas and conjunctivae were incubated at 37 °C for 30 min with anti-CD4 (FITC) and anti–IFN-γ (PE) antibodies (BD Biosciences, CA, USA), along with matched isotype controls. Fluorescence signals were acquired using a FACSCalibur system and analyzed with CellQuest [25].

Tissue lysates were prepared in buffer with protease inhibitors and centrifuged (14,000× *g*, 15 min, 4 °C). The supernatants were stored at −70 °C. TNF-α was quantified using a Luminex 200 system (Luminex, Austin, TX, USA), and the data were analyzed with xPONENT software version 4.2 [26,27].

### 2.8. Evaluation of Glycocalyx Integrity and Goblet Cell Density

After fixation, corneas were BSA-blocked and stained with Alexa Fluor 488 wheat germ agglutinin (WGA, Thermo Fisher, Waltham, MA, USA). Images were obtained via confocal microscopy, and three regions per cornea were evaluated using ImageJ (NIH) [28].

After overnight fixation (4% paraformaldehyde, 4 °C), ocular tissues were paraffin-embedded and cut into 6 μm sections. These were deparaffinized and stained with 0.5% PAS to visualize goblet cells. Brightfield images were acquired, and goblet cell densities in upper and lower conjunctivae were measured using Image-Pro software (Version 10.0.5, Media Cybernetics, Rockville, MD, USA) and normalized to 800 μm of epithelium [24].

### 2.9. Data Analysis and Statistics

One-way ANOVA with Tukey’s test was used for analysis (*p* < 0.05). The results are shown as the mean ± SD. GraphPad Prism 9 was used for plotting and statistics.

## 3. Results

### 3.1. Assessment of Cell Viability and Apoptosis Under Hyperosmotic Stress In Vitro

Preliminary experiments, including the MTT assay, performed to assess the effects of different NaCl concentrations on cell viability showed that relative to a 100 ± 4.47% cell viablity in 0 mM NaCl, 50 mM NaCl reduced the cell viability to 94.39 ± 4.46%, 100 mM NaCl reduced it to 77.08 ± 6.87%, and 500 mM NaCl reduced it to 5.58 ± 1.42%. Based on these results, 100 mM NaCl was selected for analysis, because it consistently induced a moderate level of hyperosmotic stress, making it suitable for testing the protective effects of the treatments.

**Figure 1:**
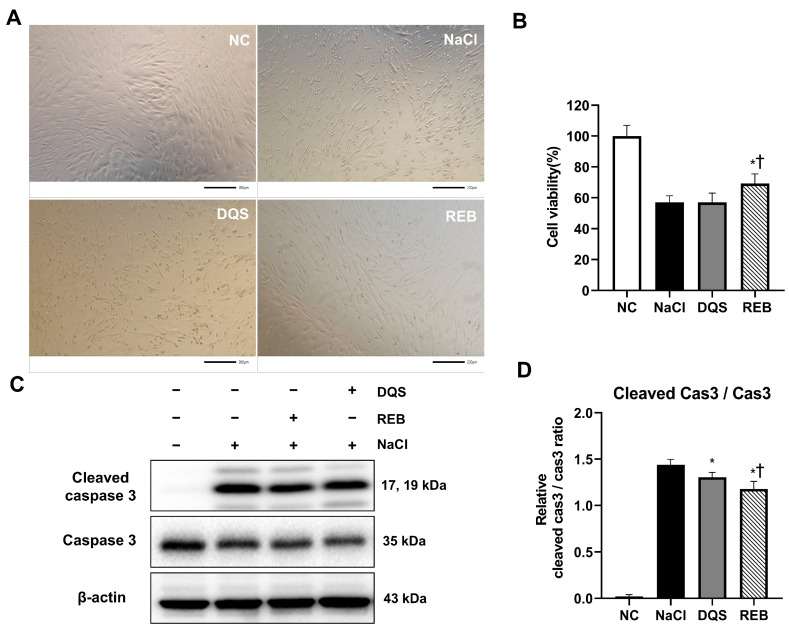
The effects of REB and DQS on cell viability and apoptosis in HCECs. (**A**) Representative images of morphological changes in HCECs. (**B**) Cell viability under hyperosmotic stress with or without REB or DQS. (**C**) Western blot analysis of cleaved caspase-3, total caspase-3, and β-actin. (**D**) Quantification of the cleaved caspase-3/caspase-3 ratio. Data are shown for the NC, NaCl, DQS, and REB groups. Each value is expressed as the mean ± SD. * *p* < 0.05 vs. the NaCl group; † *p* < 0.05 vs. the DQS group.

Under 100 mM NaCl-induced stress, representative microscopy images (Figure 1A) revealed distinct morphological changes between groups. The cells in the NC group were elongated and spindle-shaped with high density. However, cells in the NaCl group showed shrinkage, rounding, and gaps, indicating stress damage. The cells in the DQS group showed a slight improvement in cell shape and density compared to those in the NaCl group, with limited restoration. In contrast, the REB group exhibited a marked recovery of cell morphology, closely resembling that of the NC group.

The effects of REB and DQS on cell viability under 100 mM NaCl-induced stress were evaluated using the MTT assay. The results showed that the cell viability was 100 ± 6.93% in the NC group, 57.04 ± 4.31% in the NaCl group, 57.08 ± 5.95% in the DQS group, and 69.19 ± 6.34% in the REB group (Figure 1B). The cell viability in the REB group was significantly higher than that in the NaCl group (*p* = 0.0018). However, there was no significant difference in cell viability between the NaCl and DQS groups (*p* > 0.9999). Furthermore, the cell viability in the REB group was significantly higher than that in the DQS group (*p* = 0.0019).

To further assess the effects of REB and DQS on apoptosis, the expression levels of cleaved caspase-3, caspase-3, and β-actin were analyzed using Western blot analysis (Figure 1C,D). The mean cleaved caspase-3/caspase-3 ratio was 0.02 ± 0.02 for the NC group, 1.44 ± 0.06 for the NaCl group, 1.30 ± 0.05 for the DQS group, and 1.18 ± 0.08 for the REB group. Both the REB and DQS groups exhibited significantly reduced cleaved caspase-3 levels compared to the 100 mM NaCl group (*p* < 0.001 and *p* < 0.05, respectively). Furthermore, REB treatment significantly reduced the cleaved caspase-3 levels compared to DQS (*p* < 0.05).

### 3.2. Results of Clinical Evaluations

**Figure 2:**
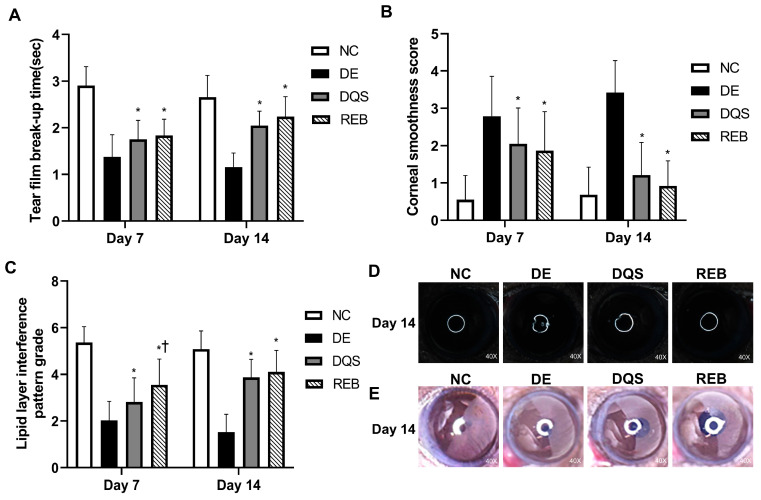
Clinical evaluations. (**A**) Mean tear film break-up time. (**B**) Mean corneal smoothness score. (**C**) Mean lipid layer interference grade. (**D**) Representative images of corneal smoothness. (**E**) Representative lipid layer patterns for the NC, DE, DQS, and REB groups at two time points (day 7 and day 14). Each value is expressed as the mean ± SD. * *p* < 0.05 vs. the DE group; † *p* < 0.05 vs. the DQS group.

On day 7, the TBUT was 2.90 ± 0.41 s for the NC group, 1.38 ± 0.47 s for the DE group, 1.75 ± 0.40 s for the DQS group, and 1.83 ± 0.35 s for the REB group. On day 14, the TBUT was 2.66 ± 0.47 s for the NC group, 1.16 ± 0.30 s for the DE group, 2.05 ± 0.31 s for the DQS group, and 2.24 ± 0.45 s for the REB group. The TBUT of the DE group was significantly lower than that of the NC group on both day 7 and day 14. Both the DQS and REB groups showed significantly improved TBUT compared to the DE group (all *p* < 0.01). However, there was no significant difference in TBUT between the two treatments (Figure 2A).

The corneal smoothness scores recorded on day 7 were 0.55 ± 0.65 for the NC group, 2.79 ± 1.07 for the DE group, 2.05 ± 0.96 for the DQS group, and 1.87 ± 1.04 for the REB group. On day 14, the scores were 0.68 ± 0.74 for the NC group, 3.42 ± 0.86 for the DE group, 1.21 ± 0.87 for the DQS group, and 0.92 ± 0.67 for the REB group. The DE group had significantly higher scores than the NC group on both days 7 and 14. Both the DQS and REB groups showed significantly reduced corneal smoothness scores compared to the DE group (all *p* < 0.01). However, there was no notable difference in corneal smoothness scores between the two interventions (Figure 2B,D).

On day 7, the lipid layer interference scores were 5.37 ± 0.67 for the NC group, 2.03 ± 0.82 for the DE group, 2.82 ± 1.04 for the DQS group, and 3.55 ± 1.11 for the REB group. On day 14, the scores were 5.08 ± 0.78 for the NC group, 1.53 ± 0.76 for the DE group, 3.87 ± 0.78 for the DQS group, and 4.11 ± 0.92 for the REB group. The DE group had significantly lower scores than the NC group on both days. On day 7, both the DQS and REB groups showed significantly higher scores than the DE group (all *p* < 0.01). In addition, the REB group exhibited significantly greater scores than the DQS group (*p* < 0.01). On day 14, both the DQS and REB groups exhibited significantly higher scores than the DE group (*p* < 0.01). Hower, there was no significant difference in lipid layer interference scores between the two treatments (Figure 2C,E).

### 3.3. ROS Levels in Corneal and Conjunctival Tissues

**Figure 3:**
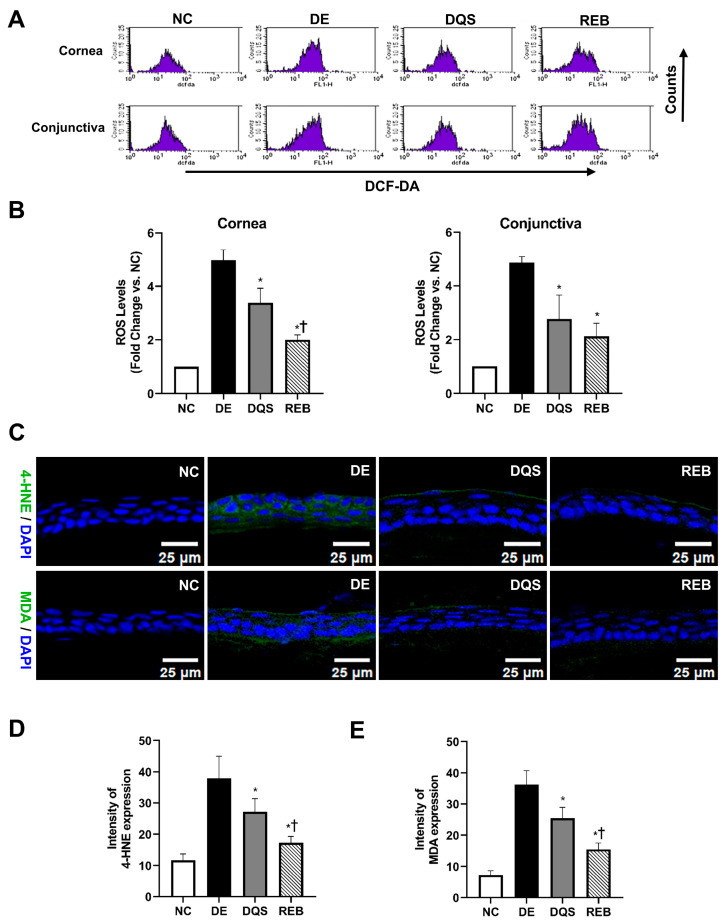
ROS and lipid peroxidation in ocular tissues. (**A**) Representative DCFDA fluorescence images. (**B**) ROS quantification in ocular surface samples (cornea and conjunctiva). (**C**) Representative immunofluorescence of 4-HNE and MDA in corneal tissue. (**D**,**E**) Quantification of 4-HNE and MDA fluorescence intensity. Data recorded after 14 days of treatment are shown for the NC, DE, DQS, and REB groups. All values are presented as the mean ± SD. * *p* < 0.05 vs. the DE group; † *p* < 0.05 vs. the DQS group.

Figure 3A depicts representative fluorescence images of the DCFDA intensity signals in the corneal and conjunctival tissues of each experimental group. The ROS levels are expressed as fold changes relative to the NC group (fold change = ROS levels of each group/ROS levels of NC group). The DE group showed significantly higher ROS levels in both the cornea (4.97 ± 0.39-fold) and conjunctiva (4.86 ± 0.23-fold, *p* < 0.01) than the NC group. However, the DQS (cornea: 3.39 ± 0.54-fold; conjunctiva: 2.77 ± 0.89-fold) and REB (cornea: 2.00 ± 0.19-fold; conjunctiva: 2.12 ± 0.49-fold) groups exhibited significantly reduced ROS levels relative to the DE group (*p* < 0.01). Moreover, the REB group showed significantly lower ROS levels in corneal tissues than the DQS group (*p* < 0.01). However, there was no statistically significant difference in the ROS levels in conjunctival tissues between the two treatments (Figure 3B).

### 3.4. Expression of Lipid Peroxidation Markers (MDA and 4-HNE) in Corneal Tissues

Oxidative stress was assessed by measuring the MDA and 4-HNE levels in the corneal tissues. The mean 4-HNE level was 11.71 ± 2.03 MFI for the NC group, 37.91 ± 7.12 MFI for the DE group, 27.19 ± 4.19 MFI for the DQS group, and 17.25 ± 2.05 MFI for the REB group. Both DQS and REB significantly decreased the 4-HNE levels compared to those in the DE group (*p* < 0.01 and *p* < 0.0001, respectively). In addition, the REB group showed significantly lower 4-HNE levels than the DQS group (*p* = 0.03, Figure 3C,D).

The mean MDA level was 7.26 ± 1.38 MFI for the NC group, 36.32 ± 4.43 MFI for the DE group, 25.43 ± 3.43 MFI for the DQS group, and 15.49 ± 2.04 MFI for the REB group. The DQS and REB groups showed significantly reduced MDA levels compared to the DE group (*p* <0.01 and *p* < 0.0001, respectively), with the REB group showing significantly lower levels than the DQS group (*p* = 0.003, Figure 3C,E).

### 3.5. Evaluation of Corneal Epithelial Apoptosis

**Figure 4:**
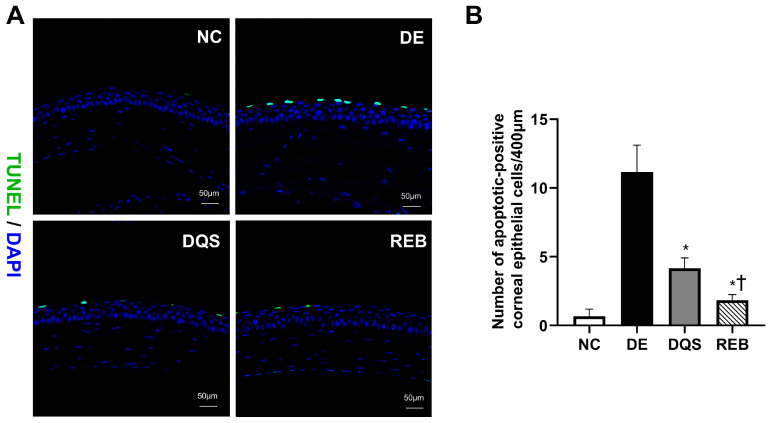
Assessment of corneal apoptosis using the TUNEL assay. (**A**) Representative TUNEL (green) and DAPI (blue) staining of corneal sections. (**B**) Quantification of TUNEL-positive cells in the NC, DE, DQS, and REB groups after 14 days of treatment. Each value is expressed as the mean ± SD. * *p* < 0.05 vs. the DE group; † *p* < 0.05 vs. the DQS group.

TUNEL-positive (green) and DAPI-stained (blue) corneal sections from each group are illustrated in Figure 4A. The average number of apoptotic cells per 400 μm of corneal epithelium was 0.67 ± 0.52 for the NC group, 11.17 ± 1.94 for the DE group, 4.17 ± 0.75 for the DQS group, and 1.83 ± 0.41 for the REB group. Both the DQS and REB groups exhibited markedly reduced apoptotic cell counts relative to the DE group (*p* < 0.0001). However, the REB group showed significantly fewer apoptotic cells than the DQS group (*p* = 0.007; Figure 4B).

### 3.6. CD4^+^ IFN-γ^+^ T Cells and Inflammatory Cytokines in the Cornea and Conjunctiva

**Figure 5:**
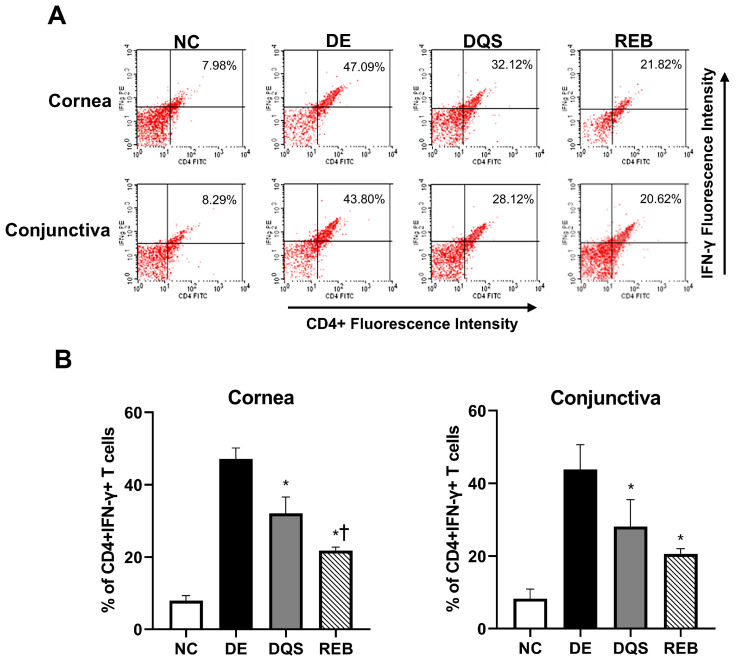
Flow cytometric analysis of CD4^+^ IFN-γ^+^ T cells in corneal and conjunctival tissues. (**A**) Representative images. (**B**) Mean percentages of CD4^+^ IFN-γ^+^ T cells in the cornea and conjunctiva after 14 days of treatment (data are shown for the NC, DE, DQS, and REB groups). Each value is expressed as the mean ± SD. * *p* < 0.05 vs. the DE group; † *p* < 0.05 vs. the DQS group.

Representative histograms of CD4^+^ IFN-γ^+^ T cell percentages in samples from the NC, DE, DQS, and REB groups are shown in Figure 5A,B. The proportion of CD4^+^ IFN-γ^+^ T cells in the corneal tissue was 7.98% ± 1.41% for the NC group, 47.09% ± 3.06% for the DE group, 32.12% ± 4.49% for the DQS group, and 21.82% ± 0.95% REB group. The proportion of cells in the conjunctiva was 8.29% ± 2.63% for the NC group, 43.80% ± 6.80% for the DE group, 28.12% ± 7.38% for the DQS group, and 20.62% ± 1.43% for the REB group. The DQS and REB groups showed a notable reduction in the proportion of CD4^+^ IFN-γ^+^ T cells in corneal and conjunctival tissues relative to the DE group (all *p* < 0.05). In addition, the REB group exhibited a significantly lower proportion of CD4^+^ IFN-γ^+^ cells in corneal tissue than the DQS group (*p* < 0.01). However, there was no statistically significant difference in the proportion of cells in conjunctival tissue between the two treatment groups. Although the TNF-α levels varied among the groups, the differences did not reach statistical significance. The mean concentrations in the cornea were as follows: 2.43 ± 1.84 pg/mg for the NC group, 5.39 ± 5.69 pg/mg for the DE group, 2.97 ± 1.49 pg/mg for the DQS group, and 4.15 ± 3.03 pg/mg for the REB group.

### 3.7. Quantification of Corneal Glycocalyx-Stained Areas and Goblet Cell Density

**Figure 6:**
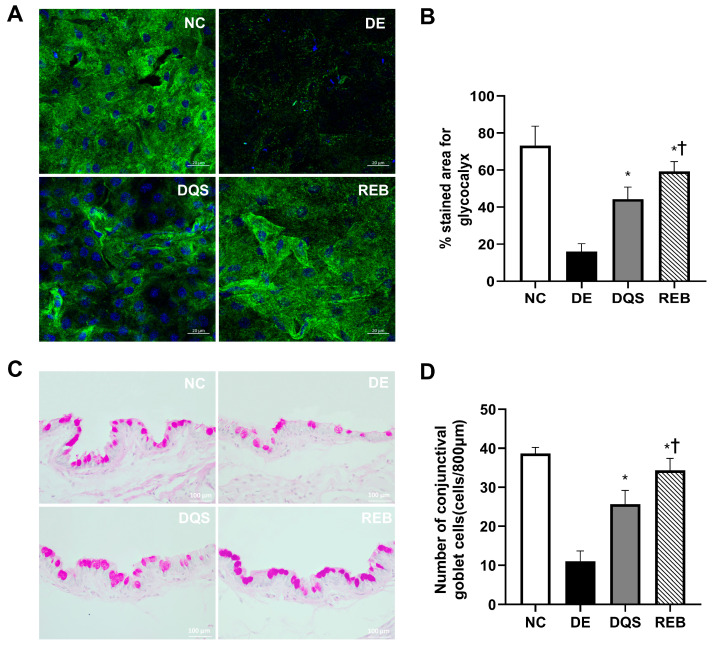
Quantification of corneal glycocalyxes and conjunctival goblet cells. (**A**) Representative confocal Z-stack images of glycocalyxes (green) and nuclei (blue). (**B**) Mean glycocalyx-stained area. (**C**) PAS-stained images of conjunctival goblet cells. (**D**) Goblet cell density in the NC, DE, DQS, and REB groups after 14 days. Each value is expressed as the mean ± SD. * *p* < 0.05 vs. the DE group; † *p* < 0.05 vs. the DQS group.

Threshold-based quantification was performed to determine the proportion of the corneal surface that showed glycocalyx-positive (green) staining (Figure 6A). The mean proportion of the stained area was 73.25% ± 10.44% in the NC group, 16% ± 4.243% in the DE group, 44.25% ± 6.55% in the DQS group, and 59.25% ± 5.32% in the REB group. The DQS and REB groups showed significant increases in the stained area compared to the DE group (*p* < 0.001 and *p* < 0.0001, respectively). However, the REB group exhibited a significantly larger stained area than the DQS group (*p* < 0.05) (Figure 6B).

Representative histological sections showing the conjunctival goblet cell density in each experimental group are shown in Figure 6C. The average goblet cell counts per 800 µm of epithelium were as follows: 38.7 ± 1.5 cells for the NC group, 11.0 ± 2.6 cells for the DE group, 25.7 ± 3.5 cells for the DQS group, and 34.3 ± 3.1 cells for the REB group. Both treatment groups (DQS and REB) showed statistically significant increases in goblet cell counts compared to the DE group (*p* < 0.001 and *p* < 0.0001, respectively). However, the number of goblet cells in the REB group was significantly higher than that in the DQS group (*p* = 0.02; Figure 6D).

## 4. Discussion

DED is caused by disruption of tear film homeostasis, a process that induces chronic inflammation and activates immune responses that accelerate epithelial damage, goblet cell loss, and apoptosis [29,30]. It has been widely reported that patients with DED show elevated IL-1β, IL-6, IL-8, and TNF-α levels in the tear film and conjunctival epithelium, which highlights the central role of these markers in promoting ocular surface inflammation and driving disease progression [31,32]. Oxidative stress plays a pivotal role in the pathophysiology of DED, because excessive ROS promotes corneal epithelial damage and inflammation. Environmental stressors, such as ultraviolet radiation, air pollution, and aging, contribute to oxidative imbalance, which leads to lipid peroxidation, damages the glycocalyx, and decreases mucin secretion. These changes compromise tear film stability and weaken the epithelial barrier, further aggravating ocular surface dysfunction [5,33,34,35,36].

Among the various treatment options for DED, artificial tears (ATs) are widely used to provide symptomatic relief. However, previous studies have demonstrated that both DQS and REB are clinically more effective than ATs in improving ocular surface parameters, including TBUT, corneal fluorescein staining scores, and Schirmer’s test results [37,38,39,40]. In addition, the roles of REB and DQS in promoting mucin secretion and stabilizing the tear film have been investigated. Nonetheless, comparative studies of the antioxidative and anti-apoptotic effects of both treatments remain limited. In the present study, we investigated the protective effects of REB and DQS beyond their mucin-secretagogue properties, particularly their roles in preserving epithelial integrity by reducing oxidative damage, inhibiting apoptosis, and maintaining goblet cell function. Specifically, we determined the therapeutic effects of REB and DQS on oxidative stress, apoptosis, and ocular surface protection in a murine DED model and a hyperosmotic stress-induced HCEC model. Our results demonstrate that REB treatment significantly reduced oxidative stress markers, including ROS levels, MDA, and 4-HNE, indicating superior antioxidative properties compared to DQS. Additionally, REB demonstrated a stronger anti-apoptotic effect than DQS, as indicated by a reduction in corneal epithelial apoptotic cells and suppressed cleaved caspase-3 expression in the REB group. Furthermore, REB significantly reduced CD4^+^ IFN-γ^+^ T cell infiltration in the cornea, which may reflect its anti-inflammatory properties and modulation of the ocular immune environment. Moreover, REB treatment increased goblet cell density and enhanced glycocalyx integrity, highlighting its potential role in preserving ocular surface stability.

Oxidative stress has been identified as a key factor in the pathogenesis of DED. Previous studies have shown that oxidative damage to corneal epithelial cells leads to lipid peroxidation, mitochondrial dysfunction, and increased apoptosis. Moreover, overproduction of ROS contributes to the upregulation of inflammatory pathways [6,7]. The results of the present study demonstrated that REB treatment effectively decreased ROS and oxidative stress markers (4-HNE and MDA), suppressed corneal epithelial apoptosis, and inhibited caspase-3 activation. Furthermore, REB enhanced cell survival under hyperosmotic stress, suggesting superior antioxidant and anti-apoptotic efficacy compared to DQS. These protective effects are consistent with findings from previous reports indicating that REB may exert its antioxidant and cytoprotective functions via the activation of signaling pathways implicated in oxidative stress regulation, including sirtuin 1, forkhead box O3a, and nuclear factor erythroid 2-related factor 2, which upregulate cellular antioxidant defenses [41]. This effect may be further enhanced by the activation of peroxisome proliferator-activated receptor gamma, which contributes to cytoprotective responses [41]. Moreover, REB has been shown to suppress ROS production and caspase-1 activation, reinforcing its anti-apoptotic activity [42]. These findings highlight the potential of REB as a therapeutic agent that can mitigate oxidative stress-induced epithelial apoptosis in DED.

In the present study, REB treatment significantly increased goblet cell density and improved glycocalyx integrity compared to DQS treatment. Although both REB and DQS function as mucin secretagogues, their mechanisms of action differ fundamentally. DQS primarily functions as a P2Y2 receptor agonist, stimulating the secretion of aqueous tear fluid and mucins from conjunctival epithelial and goblet cells, respectively. This mechanism enhances tear film hydration and lubrication, thereby contributing to the improvement of ocular surface moisture [9,43,44]. On the other hand, REB stimulates mucin-like glycoprotein production through activation of the epidermal growth factor receptor pathway, resulting in the upregulation of membrane-associated mucins (MUC1, MUC4, and MUC16). This process subsequently enhances epithelial protection and stability [45,46,47,48,49]. Our results revealed that the REB-treated goblet cells not only increased in density, but also appeared larger, indicating structural alterations associated with goblet cell function. These findings are consistent with those of previous studies, which have indicated that REB upregulates goblet cell function and mucin biosynthesis [28,50,51]. These differences suggest that REB may provide superior long-term ocular surface protection by both stimulating mucin secretion and strengthening the epithelial barrier.

This study has several limitations that should be acknowledged. First, although both in vivo and in vitro models were employed to investigate the protective effects of REB, the upstream molecular regulators and signaling pathways involved in its antioxidative and anti-apoptotic mechanisms were not fully delineated. Future studies incorporating targeted pathway analyses are warranted to deepen mechanistic understanding. Second, as this study primarily focused on short-term outcomes, further investigation is required to evaluate the long-term efficacy and safety of REB in the treatment of dry eye disease. Finally, although both DQS and REB are clinically approved secretagogues that are commonly used to treat dry eye with a short tear break-up time, they are not typically co-administered in clinical practice. The potential interaction between the two agents was not explored in this study, and remains an important subject for future investigation.

## 5. Conclusions

This study demonstrated that compared to DQS, REB provided superior protection against oxidative stress and apoptosis in a murine DED model and in hyperosmotic stress-induced HCECs. The results showed that REB significantly reduced oxidative stress markers, suppressed apoptosis, and enhanced epithelial integrity. These findings underscore the potential of REB as a comprehensive therapeutic option for patients with DED associated with oxidative stress and inflammation-induced epithelial injury, such as Sjögren’s syndrome-associated dry eye.

## Data Availability

The data supporting the findings of this study are available from the corresponding author upon reasonable request. The datasets are not publicly available due to restrictions related to internal research protocols and ethical considerations.
